# Assessing the viability of monospaced fonts for eye-movement paradigms in Arabic reading

**DOI:** 10.3758/s13428-026-03173-z

**Published:** 2026-11-12

**Authors:** Maryam A. AlJassmi, Kevin B. Paterson, Manuel Perea, Sami Boudelaa, Alia Ali, Maitha Alshaali, Alyaziah AlShamsi, Amani Mohammed, Kayleigh L. Warrington

**Affiliations:** 1 Department of Cognitive Sciences, College of Humanities and Social Sciences, United Arab, Emirates University, Al Ain, United Arab Emirates; 2 School of Psychology and Vision Sciences, University of Leicester, Leicester, UK; 3 Department of Psychology, Zayed University, Dubai, United Arab Emirates; 4 Department of Methodology and, ERI-Lectura, Universitat de València, Valencia, Spain; 5 Centro de Investigación Nebrija en Cognición, Universidad Nebrija, Madrid, Spain

**Keywords:** Arabic reading, Eye movements, Parafoveal processing, Monospaced vs. proportional fonts, Font type

## Abstract

Monospaced (fixed-width) fonts such as Courier, where each character occupies an identical unit of horizontal space, are widely used in eye-movement research in Latinate languages (Slattery, 2016). The uniformity of monospaced fonts is methodologically advantageous, allowing researchers to match the spatial width of words containing equal numbers of letters. However, their use has been avoided in Arabic research due to their rarity and perceived unnatural appearance, which changes the natural cursive interconnection between letters (Hermena et al., 2017). This imposes difficulties for researchers of Arabic reading, particularly when employing gaze-contingent change techniques, creating restrictions that impede progress to close the gap in knowledge about cursive languages. While it has been hypothesized that monospacing may interfere with cognitive processing, this has not been directly investigated. In two experiments, we examined whether monospaced and proportional fonts modulate lexical frequency effects and parafoveal preview effects during Arabic sentence reading. Fixation time measures showed similar patterns in both font types. Lexical frequency influenced word identification similarly across most measures in the two font types (Experiment 1), while a manipulation of parafoveal preview validity produced comparable effects on fixation times for target words (Experiment 2). Across experiments, monospaced fonts produced shorter but more frequent fixations than proportional fonts: a trade-off that mirrors effects previously reported in Latinate languages. Given the methodological advantages for gaze-contingent paradigms, these findings establish monospaced fonts as a readable and suitable option for Arabic reading research, facilitating tighter spatial control and cross-script comparability.

Monospaced (fixed-width) fonts, such as those from the Courier family, assign identical horizontal space to every character. Fixed width is achieved by compressing wider letters (e.g., m) and stretching or adding space around narrower letters (e.g. l) so that the spatial width of all letters and interword spaces is identical (e.g., compare ml with ml). This uniformity makes word length directly proportional to letter count, simplifying analyses of word length effects, saccade targeting, and the definition of interest areas (Rayner et al., 2010; Slattery, 2016). Critically, they are especially useful for gaze-contingent paradigms such as the boundary technique (Rayner, 1975), where small differences in word width can unintentionally reveal the presence of display changes. Because proportional fonts vary in character width, any changes may alter line length and make such changes more noticeable. Although proportional fonts dominate everyday print (Arditi et al., 1990; Beldie et al., 1983), monospaced fonts offer methodological advantages that have made them the standard in eye-movement research on Latinate scripts (Hautala et al., 2011; Slattery, 2016).

The use of monospaced fonts in psycholinguistic research is considered acceptable because, in Latinate languages, differences between monospaced and proportional fonts are minimal, and monospacing does not appear to disrupt linguistic processing. While some early studies reported faster reading speeds for proportional fonts (Arditi et al., 1990; Beldie et al., 1983; Payne, 1967), other studies found no such advantage (Gould et al., 1987; Muter et al., 1982). Conversely, monospaced text may support faster reading at smaller font sizes, possibly due to the uniform spacing reducing visual crowding (Arditi et al., 1990; Mansfield et al., 1996). More recent eye-movement studies suggest that word identification and parafoveal processing are largely equivalent across monospaced and proportional font types, with only minor differences in eye-movement behaviour (Hautala et al., 2011; Rayner et al., 2010; Slattery, 2016; Slattery & Rayner, 2010). Rayner et al. (2010) found comparable reading rates and perceptual span size, but some differences in fixation patterns. Specifically, fixation times were generally longer for proportional fonts, whereas monospaced fonts elicited a higher number of fixations. This pattern was attributed to a processing trade-off: wider average spatial width of letters in monospaced fonts means that fewer letters fall within foveal vision, thus requiring more fixations to complete encoding. By comparison, proportional fonts enable readers to process text with fewer fixations, although fixation durations may increase because more letter information is encoded per fixation. Despite these differences, both font types are highly readable and suitable for use in reading research (Josephson, 2008). Crucially, this body of evidence enables researchers working in Latinate languages to take advantage of the methodological benefits of monospaced fonts without compromising ecological validity or reading performance.

While extensive eye-movement research has been conducted in Latinate languages, and English in particular, other writing systems have received considerably less attention. Indeed, despite increasing recognition that overreliance on English hinders cognitive science (Blasi et al., 2022), there remain marked disparities in the countries and languages in which eye-movement research on reading takes place (Angele & Duñabeitia, 2024). As of April 2024, among documents in the Scopus eye-movement literature, 47.3% list affiliations in Europe, 24.3% in the United States, and 6.3% in China (the increase in Chinese research represents the only notable change in the literature landscape over the last 20 years). Similarly, a bibliometric analysis of eye-tracking studies of reading found that investigations of English reading accounted for 57.5% of papers published between 2000 and 2018 (Siegelman et al., 2022). This means that the majority of research still originates from a small number of countries and is conducted in only a small number of languages.

Comparatively little research has examined Semitic scripts such as Arabic, despite this script having unique visual and linguistic characteristics that could meaningfully inform our understanding of written language processing (AlJassmi et al., 2021a). Briefly, Arabic is read from right to left (Jordan et al., 2014) and uses a non-concatenative morphology where words are formed through the integration of root consonants conveying core meaning and pattern vowels encoding grammatical information (Abu-Rabia, 2002; Boudelaa & Marslen-Wilson, 2001, 2004; Tibi et al., 2020). The cursive script connects most interword letters (Alluhaybi & Witzel, 2020; Khateb et al., 2013, 2014), and letter forms are highly position-dependent, changing shape depending on their word position (Boudelaa et al., 2019; Carreiras et al., 2012), introducing additional visual variability. Many letters are visually similar, distinguished primarily by small diacritical dots (AlJassmi & Perea, 2024; Perea et al., 2016a, 2016b). The script also has high informational density, with a large amount of linguistic information conveyed through compact visual forms (AlJassmi et al., 2021b). These features place unique demands on reading, shaping attention allocation, visual discrimination, and reliance on contextual cues—factors likely to be reflected in eye-movement metrics. In addition to Arabic itself, the script is used in many non-Semitic languages, including Persian (Indo-European), Uyghur (Turkic), and others, broadening its global relevance and introducing further variation in linguistic structure. As such, Arabic offers a theoretically rich and underexplored opportunity for advancing models of written language processing.

Importantly, in those few studies that have examined reading in Arabic, monospaced fonts have been avoided due to their rarity and perceived unnaturalness (Hermena et al., 2015, 2017; see Fig. 1), despite a lack of direct evidence for this. Unfortunately, this poses numerous challenges for researchers studying Arabic reading due to the lack of a consistent correspondence between the number of letters in a word and its physical length. This includes complicating the use of gaze-contingent techniques. With proportional fonts, the spatial widths of target words and preview masks cannot be easily matched, requiring manual adjustments that increase complexity and risk confounding fixation and saccade measures (Hermena et al., 2017). Given the already limited resources to aid program development and stimuli generation for Arabic studies, this additional challenge imposed by the requirement to use proportional fonts increases workload and the risk of inconsistencies, making the process significantly more onerous. Therefore, more fundamentally, this methodological disadvantage is also a matter of research equity, as it slows efforts to close “the eye-tracking gap” (Angele & Duñabeitia, 2024, p. 2) in knowledge about cursive languages and inhibits cross-language comparisons because differences arising from font characteristics are not well understood. Testing the viability of monospaced fonts is therefore critical for both methodological and theoretical advancement in the field. Our approach aims not only to evaluate their cognitive effects on reading processes, but crucially, to expand the range of tools and methodologies available for Arabic reading research.

**Fig. 1:**
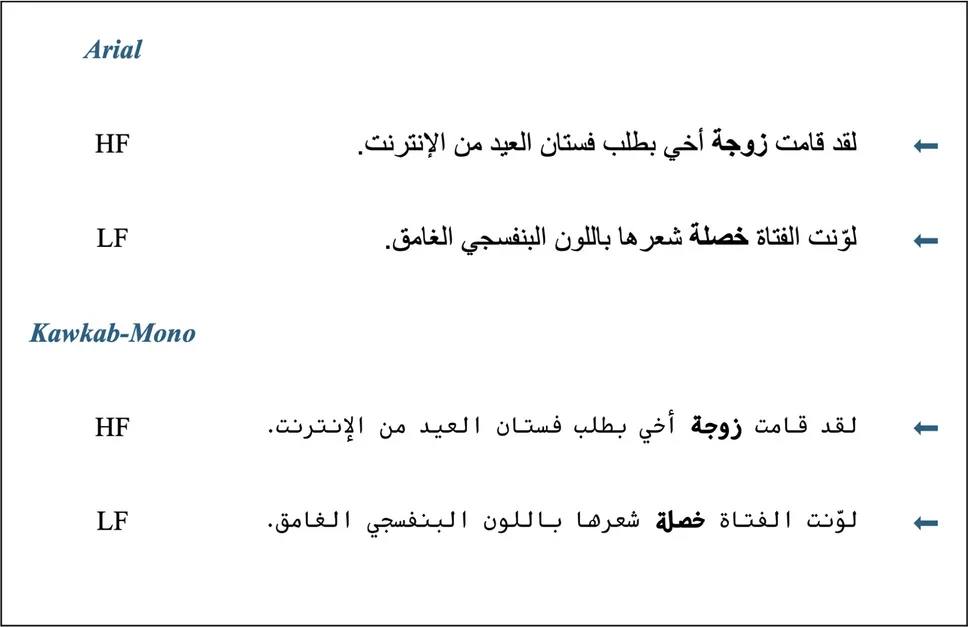
Example stimulus sentences shown in a proportional font (Arial) and a monospaced font (Kawkab-Mono). The four-letter target is shown in bold; HF = high-frequency, LF = low-frequency. Reading proceeds from right to left. Marking was not shown during the experiment. Translations: HF: *My brother’s wife ordered a ****dress**** for Eid from the Internet.* LF: *The girl dyed a ****strand**** of her hair a dark purple*

There are several reasons why monospaced fonts have been considered unsuitable for Arabic reading research. Monospaced fonts may disrupt Arabic’s natural flow and connectivity, changing the interconnection between letters by either lengthening ligatures or increasing inter-letter spacing (Alluhaybi & Witzel, 2020; Khateb et al., 2013, 2014; Roldán et al., 2018; Taha et al., 2013). In English, monospacing increases spacing systematically, creating a uniform effect throughout the text as each letter is distinctly separate (Cohen et al., 2008). In contrast, Arabic script ranges from fully connected letters in words (e.g., كلمة) to semi-connected (e.g., ساعة) and, occasionally, letters that do not connect at all (e.g., رداء). The impact of monospacing in Arabic therefore varies depending on the structural properties of each word, such that the effect is not uniform. Disruption of inter-letter connections might increase cognitive load and impede accurate word identification (Hermena et al., 2017). Moreover, lengthening ligatures or increasing spacing can also alter the overall shapes of words, potentially making it more difficult for readers to recognize words based on their typical visual patterns (see Roldán et al., 2018). Some researchers have gone so far as describing the appearance of monospaced fonts in Arabic as “extremely unnatural” (Hermena et al., 2015, p. 503). Despite these conjectures about the potential impact of monospacing, no research has directly investigated the processing of monospaced versus proportional fonts in Arabic or established how such effects might influence methodological suitability for experimental reading research.

Monospaced fonts do indeed receive limited use in Arabic. Computer programmers might use monospaced fonts when writing code that includes Arabic strings, leading to the development of Arabic monospaced fonts for this purpose (Kawkab-Mono, Arif, 2015), but this is a niche use case. In contrast, proportional fonts are preferred in digital media, print, education, and professional settings. This frequency of exposure is an important consideration, as fonts that are less commonly encountered are generally read less efficiently than common fonts (Beier & Larson, 2013; Seymour & Jack, 1978). However, only a few studies have examined typographic influences on Arabic reading (Ganayim & Ibrahim, 2013; Jordan et al., 2017), and to our knowledge, only one has directly compared monospaced and proportional fonts. Alotaibi (2007) investigated reading aloud performance for Arabic text in Times New Roman (proportional font) and Courier (monospaced font) at three font sizes (8, 10, 12 pt) among both normally sighted and visually impaired readers. At smaller print sizes, reading times were comparable across fonts, but at larger sizes, Times New Roman yielded faster reading speeds. However, because oral reading slows processing compared with silent reading (Anderson & Swanson, 1937; Ashby et al., 2012; Inhoff et al., 2011; Søvik et al., 2000), it remains unclear whether similar effects emerge under silent reading conditions. Moreover, the mechanisms underlying observed effects remain undetermined, leaving the key question of methodological viability unanswered.

Given the sparse prior research, it is important to examine font-type effects systematically, as understanding these effects has theoretical, practical, and methodological significance. Such research would contribute to the broader theoretical understanding of how typographical factors (i.e., the visual and spatial characteristics of text) influence reading processes. More practically, our findings may inform font selection for Arabic texts, with possible implications for improving readability and comprehension in digital and print media. The present experiments address the methodological challenges posed by the use of proportional fonts, and in doing so contribute to the advancement of eye-movement research methodology. This work provides a foundation for designing more effective reading experiments in Arabic and offers insights for improving methodological rigor and generalizability of future research, thereby contributing meaningfully to “closing the gap” in reading research.

Because of its characteristics, monospacing may impair reading efficiency in Arabic, as it affects properties that are crucial for lexical identification, compromising the visual harmony of the text, and potentially disrupting the process of word recognition. However, despite widespread assumptions that monospaced fonts are unsuitable for Arabic, this claim remains untested. It is also plausible that certain features of monospacing, such as reduced crowding from increased lines/spaces between letters, may aid letter segmentation and identification (Alluhaybi & Witzel, 2020; Alotaibi, 2007), potentially providing a readability advantage in this visually dense script. This raises important and previously unexplored questions about how monospacing actually influences Arabic reading. To empirically test the assumption that monospaced fonts in Arabic disrupt core reading processes, we conducted two eye-tracking experiments during sentence reading: Experiment 1 focused on foveal lexical processing, and Experiment 2 focused on parafoveal preview. Thus, these two experiments address processes central to word recognition and fluent reading, and at the same time examine factors that are relevant for assessing the methodological viability of using monospaced fonts in Arabic reading research. The specific predictions are outlined in the corresponding sections for each experiment.

## Experiment 1

Experiment 1 examined foveal lexical processing in Arabic using a well-established index of lexical processing efficiency, the word frequency effect. This refers to the phenomenon whereby words with higher written usage frequency are processed more quickly and skipped more frequently than lower-frequency words (see Rayner, 2009; see also Brysbaert et al., 2018, for review). Skipping is therefore informative for models of eye-movement control (Rayner, 2009). In the E-Z Reader model (Reichle et al., 2003), earlier lexical completion of high-frequency words in the parafovea increases the probability that the eyes skip them, as saccade programming advances to the following word. Theoretically, the word frequency effect shows that word identification is modulated by lexical properties. As such, it is widely used to test how experimental manipulations, including font characteristics, influence word processing during reading (e.g., Slattery & Rayner, 2013).

Participants in our experiment read sentences containing either high- or low-frequency target words, presented in a monospaced font designed for Arabic (Kawkab-Mono) or a commonly used proportional font (Arial). We expected the ubiquitous word-frequency effect when reading in both fonts. More importantly, we asked whether the monospaced font modulates the magnitude of word-frequency effects. On the one hand, monospaced fonts may disrupt word recognition, leading to longer reading times, more fixations, and reduced skipping. This prediction reflects the assumption that increased spatial width of words compromises visual harmony and would impair the natural flow of Arabic reading. In this scenario, the word-frequency effect would be larger in the monospaced font. These expectations are informed by previous research with uncommon fonts in Latinate-language research (Rayner et al., 2010; Slattery & Rayner, 2010).

On the other hand, an alternative (non-exclusive) possibility follows from the perceptual characteristics of Arabic. Given that the script is visually dense, monospacing may reduce crowding between letters, facilitating word identification (Alluhaybi & Witzel, 2020). In this scenario, the two fonts (proportional and monospaced) could produce similar reading times and word-frequency effects, or monospacing might even reduce the word-frequency effect. The present experiment, therefore, provides a first step in testing whether monospacing fonts influence lexical processing in Arabic, and tests the widespread assumption that monospaced fonts impair efficient reading.

### Method

#### Participants

Forty-four fluent native Arabic speakers aged 18–24 years *(M* = 20 years), all female (the lab is located on an all-female campus), voluntarily participated in the study or in exchange for a 30 AED voucher. Participants with normal or corrected-to-normal vision were recruited from UAE University and were naïve regarding the purpose of the experiment. Data from an additional two participants were excluded from the analysis due to scoring below 80% on comprehension questions. We used Hermena et al. (2019) as a reference to determine our sample size. In their Experiment 1, Hermena et al. found a clear effect of orthographic frequency during Arabic sentence reading with 42 participants and 15 items per condition. As we were primarily interested in interactive effects (font type × frequency), we aimed to at least double the number of observations per condition and so increased the number of items from 15 to 40, resulting in a total of 1,760 observations per condition. This choice follows the recommendation by Brysbaert and Stevens (2018) for mixed-effects models in psycholinguistics.

#### Stimuli and design

The experimental stimuli consisted of 160 four-letter target words, comprising 147 nouns and 13 adjectives. Half of these words had high lexical frequency (*M* = 75.6, *SD* = 152.1 counts per million) and half had low lexical frequency (*M* = 1.1, *SD* = 0.5 counts per million; (79.002) = 4.38, *p* < .001) according to the Aralex database (Boudelaa & Marslen-Wilson, 2010). Each item was a single sentence containing one target word (see Fig. 1 for examples). Frequency was manipulated between items (80 high-frequency, 80 low-frequency words), and no target appeared more than once. Each target word was embedded in a sentence consisting of 7 to 10 words (*M* = 7.9), always preceded by at least two words and followed by at least two words, ensuring the target word consistently appeared near the middle of the sentence. Pre-target words had a length of 4 to 10 letters (*M* = 5.3). Each participant first read six practice sentences, and then the 160 experimental sentences. To assess predictability, a cloze task was conducted with 26 participants who did not take part in the eye-movement study. The results showed that the target words were not predictable from the sentence context (*M* = 0.6% correct completions, *SD* = 2.6%). To evaluate plausibility, a separate norming task was conducted with another group of 26 participants in which participants rated sentence plausibility on a 5-point Likert scale, where 1 indicated highly implausible and 5 indicated highly plausible. This task confirmed that the target words were highly plausible within the sentence context (*M* = 4.4, *SD* = 0.25), although ratings were slightly higher for high-frequency than low-frequency words (high; *M* = 4.45, low; *M* = 4.37), *F*(1, 158) = 4.19, *p* = .042.

#### Apparatus

Eye movements were recorded using an EyeLink 1000 Plus (SR Research Ltd.) desktop-mounted eye-tracker with a high-speed 35-mm lens, at a sampling rate of 1,000 Hz. Right eye gaze location was recorded each millisecond during binocular viewing. Sentences were displayed from a distance of 80 cm on a high-definition 24-inch BenQ monitor, with a 1,920 × 1,080-pixel resolution. The two fonts, Arial (proportional) rendered in size 16.5 and Kawkab-Mono (monospaced) rendered in size 11, were matched in terms of the percentage of black pixels (*p* = 0.8). However, there was a difference in terms of degrees of visual angle, with monospaced fonts always being wider (proportional = 0.2°, monospaced = 0.3° per letter).

#### Procedure

Participants were tested individually. Before the start of the experiment, each participant was instructed to read normally and be prepared to answer some comprehension questions. To ensure accurate tracking of their eye movements, a 3-point horizontal calibration procedure was employed for the eye-tracker, with the aim of maintaining a spatial error below .30°. Calibration accuracy was evaluated prior to each trial, and if necessary, recalibration was performed to maintain the desired low spatial error. At the onset of each trial, a fixation square, equivalent in size to a character space, appeared on the right side of the screen, as Arabic text is read from right to left. Once the participant fixated this square, it was replaced by the initial character of the sentence. Participants indicated they had finished reading each sentence by pressing a key on a response box. The sentence then disappeared and was replaced on 30% of the trials by a two-alternative, forced-choice comprehension question. Participants answered this question by pressing a “yes” or “no” response key. For each participant, the experiment lasted approximately 30 min.

#### Analyses

Following standard procedures, fixations shorter than 80 ms or longer than 1,200 ms were discarded (5.3% of fixations). For continuous measures, we also removed observations ≥ 2.5 standard deviations from the mean of each participant in each condition (accounting for < 3% of observations for each measure). The remaining data were analysed using the lme4 package (version 1.1–35.4; Bates et al., 2015) in R (R Core Team, 2023). Models were fit using the BOBYQA optimizer, with iterations set at 100,000 (Powell, 2009). Sentence-level analyses were computed to examine overall reading times and characterize the patterns of eye-movement behaviour for the two font types. These comprised sentence reading time, average fixation duration, number of fixations, progressive saccade length, and regression rate. For target words, we computed four measures to explore initial, first-pass word processing, which refers to the initial processing of a word prior to a saccade to its left (in Arabic) or a regressive eye movement from that word. These measures comprised word-skipping probability, first-fixation duration (FFD, the duration, in milliseconds, of the first fixation on a word during first-pass reading), single-fixation duration (SFD, the duration, of the first fixation when only a single fixation was made), and gaze duration (GD, the sum of all first-pass fixations on a word). For all analyses, participants and stimuli were treated as crossed random effects and font type was treated as a fixed effect. For target-word analyses, frequency, plus all interactions between font type and frequency were additionally included as fixed effects. Frequency and font type were contrast coded (− 0.5, 0.5) to compare the means of the two levels. For all models, we started with a maximal random effects structure, specifying random intercepts and slopes of the fixed factors across both participants and items (Barr et al., 2013). In cases of non-convergence (e.g., convergence or singularity warnings), we simplified the random structure only as required to obtain a converging model.^1^ The structure was trimmed by first removing the interaction between random slopes (in the case of target-word analyses). Where necessary, correlations between random effects were removed. Random slopes were removed sequentially, first removing slopes that were nearly or perfectly correlated with others and then those explaining the least variance, until the model converged. Final converging model structures for target-word measures are listed in full below the corresponding statistical output tables.

Analyses of raw and log-transformed data were undertaken. The statistical pattern for continuous measures was consistent across raw and log-transformed data, and so for ease of interpretation, we focus on the analysis of raw data. For all analyses, effects were considered statistically significant at *p* < .05 (equivalent to t/z values of <  − 1.96 or > 1.96). For linear mixed-effects models, *p*-values were obtained using the lmerTest package, while *p*-values for generalized linear mixed-effects models were based on Wald *z* tests.

### Results

Means and standard errors for sentence-level measures are shown in Table 1, with statistical effects summarized in the text. Means and standard errors for target-word measures are in Table 2, and statistical output in Table 3.

**Table 1:** Means and standard errors (in parentheses) for sentence-level measures in Experiment 1 and Experiment 2. Reading time measures given in milliseconds (ms), saccade length in characters, and regression rate as the percentage of saccades that were regressive (i.e., to earlier parts of the text). For all means, standard errors are calculated across participant variance (such that in Experiment 1*N* = 44 and in Experiment 2*N* = 40)

	Sentence reading time	Average fixation duration	Number of fixations	Progressive saccade length	Regression rates
Experiment 1					
Arial	2,920 (207)	241 (6)	10.4 (0.5)	4.92 (0.74)	16.57 (2.5)
Kawkab-Mono	2,963 (198)	228 (5)	11.1 (0.5)	4.83 (0.73)	15.18 (2.3)
Experiment 2					
Arial	3,435 (183)	262 (7)	11.8 (0.5)	4.47 (0.18)	13.89 (2.46)
Kawkab-Mono	3,478 (184)	246 (6)	12.6 (0.5)	4.45 (0.18)	14.01 (2.14)

**Table 2:** Means and standard errors (in parentheses) for target-word measures in Experiment 1

	Arial		Kawkab-Mono	
	High frequency	Low frequency	High frequency	Low frequency
Word skipping	0.15 (.02)	.12 (.01)	0.13 (.02)	.10 (.02)
Single-fixation duration	256 (8)	287 (14)	248 (8)	278 (16)
First-fixation duration	243 (6)	264 (10)	237 (7)	261 (11)
Gaze duration	287 (12)	317 (17)	274 (9)	321 (19)

**Table 3:** Target-word analyses for Experiment 1, showing fixed effect estimates (*b*), standard errors (*SE*), and *t*/*z*-values for frequency (high, low) and font (Arial, Kawkab-Mono) on word skipping, single-fixation duration (SFD), first-fixation duration (FFD), and gaze duration (GD). For convenience, estimates with an absolute value of < 0.01 are reported as 0.00

Fixed effect	*b*	*SE*	*t*/*z*	*p* value
Word skipping				
Intercept	− 2.37	0.16	− 14.66	< .001
Frequency	− 0.30	0.14	− 2.19	.029
Font	− 0.27	0.08	− 3.52	< .001
Frequency × font	0.00	0.15	0.00	.998
SFD				
Intercept	267.96	10.80	24.82	< .001
Frequency	31.74	9.10	3.49	< .001
Font	− 6.15	4.00	− 1.54	.133
Frequency × font	− 1.50	5.82	− 0.26	.797
FFD				
Intercept	251.06	8.05	31.18	< .001
Frequency	21.73	6.16	3.53	< .001
Font	− 4.09	3.33	− 1.23	.226
Frequency × font	4.50	5.06	0.89	.375
GD				
Intercept	299.63	13.83	21.67	< .001
Frequency	39.12	10.37	3.77	< .001
Font	− 3.55	3.90	− 0.91	.368
Frequency × font	18.34	7.23	2.54	.011

The relevant maximal structure for these models is (depvar ~ freq*font + (1 + freq*font |pp) + (1 + font|stim). Final converging model structures were as follows: Word Skipping = glmer(depvar ~ freq*font + (1 + freq |pp) + (1 |stim), data = datafile, family = binomial, control = glmerControl(optimizer = "bobyqa", optCtrl = list(maxfun = 100,000))); SFD = lmer(depvar ~ freq*font + (1 + freq + font |pp) + (1 + font |stim), data = datafile_SFD, control = lmerControl(optimizer = "bobyqa", optCtrl = list(maxfun = 100,000))); FFD = lmer(depvar ~ freq*font + (1 + freq + font |pp) + (1 + font |stim), data = datafile_FFD, control = lmerControl(optimizer = "bobyqa", optCtrl = list(maxfun = 100,000))); GD = lmer(depvar ~ freq*font + (1 + freq + font || pp) + (1 | stim), data = datafile_GD, control = lmerControl(optimizer = "bobyqa", optCtrl = list(maxfun = 100,000)))

#### Sentence-level analyses

There was no significant effect of font on sentence reading times (*b* = 46.27, *SE* = 34.37, *t* = 1.35, *p* = .185). However, average fixation durations were significantly shorter for the monospaced than for proportional font (*b* =  − 12.45, *SE* = 1.21, *t* =  − 10.31, *p* < .001), but readers made more fixations overall for the monospaced font than proportional font (*b* = 0.72, *SE* = 0.12, *t* = 6.28, *p* < .001). This pattern suggests a trade-off that mirrors the pattern observed by Rayner et al. (2010) in English. When calculated in terms of number of characters traversed, progressive saccades were slightly longer for the proportional than for the monospaced font (*b* =  − 0.09, *SE* = 0.04, *t* =  − 2.17, *p* = .034) and the rate of regressive saccades was slightly higher for the proportional than for the monospaced font (*b* =  − 1.41, *SE* = 0.37, *t* =  − 3.79 *p* < .001).

#### Target-word measures

**Fig. 2:**
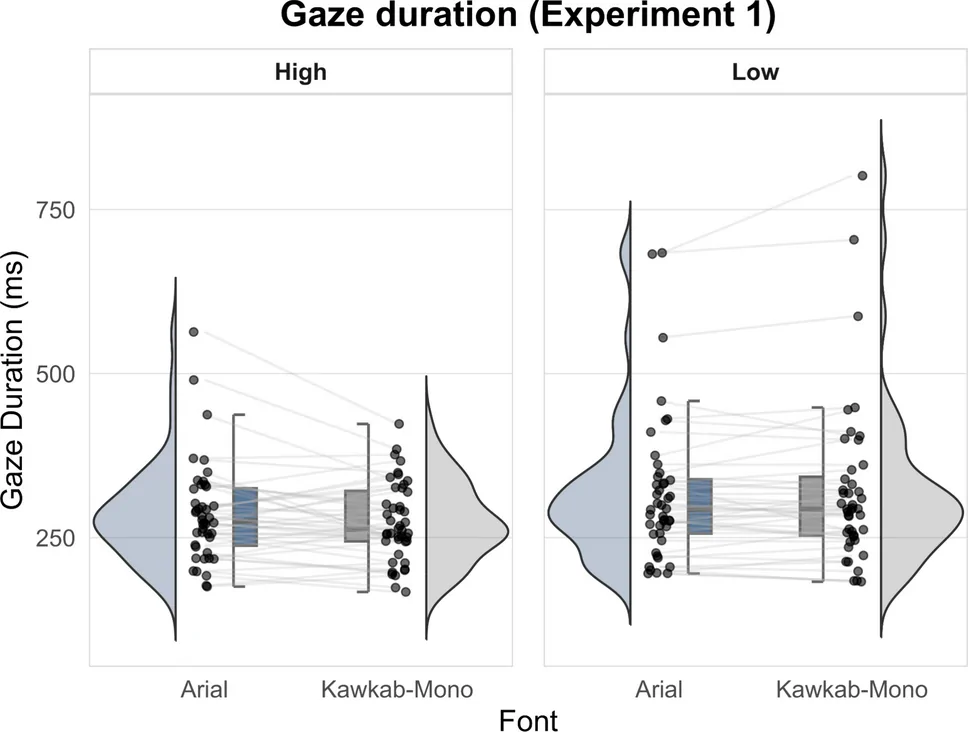
Raincloud plots of gaze duration (ms) in Experiment 1 as a function of font (Arial vs. Kawkab-Mono) and word frequency (high vs. low). Each distribution includes the probability density (half-violin), the median and interquartile range (boxplot), and individual participant means (dots). Gray lines connect the same participant across fonts within each frequency condition

FFD, SFD, and GD all showed significant effects of frequency, with longer fixations for low-frequency than high-frequency words. There were no significant effects of font in any reading time measure. In FFD and SFD, there was no evidence of an interaction between font and frequency. However, there was a significant interaction for GD, with a larger effect of frequency for the monospaced font (47 ms) compared with the proportional font (30 ms). Inspection of condition means suggests that this pattern does not reflect increased processing difficulty in the monospaced font. Instead, gaze durations for high-frequency words were numerically shorter in the monospaced font, while gaze durations for low-frequency words were similar across fonts (a Raincloud plot shows the distribution of gaze durations in Fig. 2). A significant main effect of font was found for word skipping, with a lower skipping rate for the monospaced font (11.5%) than the proportional font (13.9%). There was also a main effect of frequency, with skipping rates lower for low-frequency (11.3%) than for high-frequency (14.1%) words. No interaction was found between font and frequency.

### Discussion

In the present experiment, reading times did not differ between the two font types, indicating that overall reading efficiency is relatively robust to font variations (see Minakata & Beier, 2021). However, we found subtle but systematic differences in eye-movement behaviour. Fixation durations were longer for proportional fonts, while monospaced fonts attracted a higher number of fixations, mirroring the pattern reported in English by Rayner et al. (2010). Monospacing increases the average amount of space around each letter, which reduces visual crowding and allows information within the fovea to be processed efficiently, leading to shorter fixations. At the same time, this increased spacing extends the spatial width of words, so fewer letters fall within the fovea on a single fixation. These opposing factors create a trade-off between fixation duration and number of fixations, cancelling out differences in overall reading times. The present results indicate that this trade-off may also occur for Arabic reading. Importantly, this pattern is not unique to monospacing. Similar effects have been reported in Latinate studies when inter-letter spacing is increased in proportional fonts (e.g., Perea et al., 2016a, 2016b). Thus, the differences between font types can be best explained by spatial width rather than any disruption specific to monospaced fonts.

For target words, high-frequency words were processed more efficiently than low-frequency words across both font types, demonstrating the expected frequency effect and replicating Hermena et al. (2019). Unlike Hermena et al., we also observed lower skipping rates for low-frequency words. Given the existing debate around whether Arabic readers modulate skipping behaviour on the basis of the lexical properties of words (see also AlJassmi et al., 2021b), this issue warrants further investigation.

Crucially, during initial processing, word-frequency effects were largely similar for the two fonts. The only interaction emerged in gaze duration, where the word-frequency effect was larger in the monospaced condition. Inspection of the means suggests that this reflected numerically shorter gaze durations for high-frequency words in the monospaced font, whereas low-frequency words showed similar gaze durations across fonts. This may indicate that, counter to expectations, monospacing may support more efficient processing of high-frequency words. Because this pattern was confined to a single measure, it should be interpreted cautiously; nonetheless, it is inconsistent with the claim that monospacing disrupts lexical processing.

**Fig. 3:**
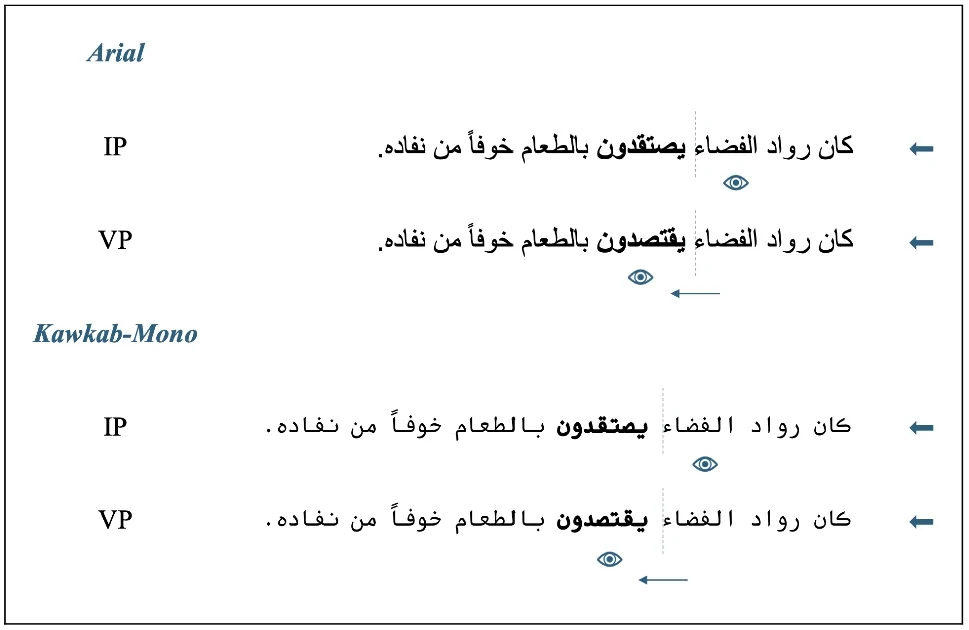
Boundary paradigm for Arabic reading. While the reader fixates the pre-target word (eye icon), the upcoming target position shows a valid preview (VP) or an invalid preview (IP). When gaze crosses the boundary, the preview is replaced by the target. Reading proceeds from right to left. Examples are shown in Arial and Kawkab-Mono. Annotations were not visible during the experiment. Translation (VP form): *The astronauts were ****economizing**** with food for fear it would run out*

Overall, our results indicate that, as in Latinate languages, font type may shape some aspects of eye-movement control, but we found little evidence that monospacing disrupts foveal lexical processing. What remains unclear, however, is how font influences the extraction of information from upcoming words. To address, this issue, we designed Experiment 2, which used the boundary paradigm (Rayner, 1975), the standard method for examining parafoveal processing in reading (see Rayner, 2009, for review), under the two font conditions (see Fig. 3).

## Experiment 2

Experiment 2 examined whether monospaced and proportional fonts differentially affect parafoveal processing in Arabic reading, using the boundary paradigm (Rayner, 1975), a gaze-contingent display change technique. In this paradigm, a target word is initially presented either as a valid preview (normally displayed) or an invalid preview (replaced with a pseudoword) prior to fixation. When the reader’s gaze crosses an invisible boundary just before the target, the preview is replaced with the actual word. This method allows researchers to measure the parafoveal preview benefit (i.e., the reduction in fixation times on target words following a valid versus an invalid preview; Parker et al., 2017; Rayner, 1998, 2009; Schotter et al., 2012).

Parafoveal processing is reduced when reading is more difficult (e.g. Pan et al., 2021; Schotter et al., 2012) or when visual complexity is high (e.g. Xie et al., 2019). This is particularly relevant for Arabic, where key morphological information may be distributed throughout the word and the most important root letters for lexical identification are not necessarily located at the beginning (Abu-Rabia, 2002; AlJassmi et al., 2021a; Boudelaa et al., 2023, 2004; Tibi et al., 2020). Indeed, in our stimuli, the root letters in the target words never appeared in the initial position but were preceded by one to three letters (*M* = 1.2). Because monospaced fonts increase letter width, root information may appear at greater parafoveal eccentricity, potentially reducing the parafoveal preview benefit (Blanchard et al., 1989; for a review, see Schotter et al., 2012).

In Experiment 1, we found reduced word skipping for words in a monospaced font, and this reduced skipping may be indicative of more limited parafoveal processing. Indeed, this aligns with Hermena et al.’s (2017) findings indicating that word skipping in Arabic reading is primarily influenced by spatial extent, rather than number of letters; wider words (e.g., شمس *sun*) are less likely to be skipped than narrow words (e.g., قلم *pen*), even when they contain the same number of letters. Thus, words presented in monospaced fonts may fail to receive sufficient parafoveal preview to support skipping. On the other hand, in Latinate languages, proportional fonts with wider letter shapes have been shown to improve letter recognition in the parafovea by reducing crowding (Oderkerk & Beier, 2022). It is possible, therefore, that the wider spacing in monospaced fonts could also support parafoveal processing in Arabic.

An additional motivation for the current experiment is that the challenge posed by proportional fonts is particularly relevant from a methodological perspective for assessing the viability of monospaced fonts. In boundary experiments, it is critical to match the spatial width of previews and targets, which is particularly challenging in proportional fonts. Therefore, if monospaced fonts allow for normal parafoveal preview, they may provide an easier and more controlled alternative for implementation of gaze-contingent manipulations in Arabic reading.

In sum, if monospaced fonts impair parafoveal processing by increasing the eccentricity of root letter information or disrupting spatial relationships between letters, we would expect a reduced or absent preview benefit in the monospaced font. Conversely, if the increased spacing of monospaced fonts reduces visual crowding and supports efficient parafoveal processing, the preview benefit should be preserved. Thus, Experiment 2 provides a critical test of whether monospaced fonts disrupt or maintain parafoveal processing in Arabic reading, providing a key test of their methodological viability for gaze-contingent paradigms.

### Method

#### Participants

Forty fluent native Arabic speakers aged 19–28 years (*M* = 21 years), all female, voluntarily participated in the study. Participants were recruited from Zayed University, UAE. Participants had normal or corrected vision, assessed using a Bailey–Lovie Eye Chart (Bailey & Lovie, 1980), and were naïve regarding the purpose of the experiment. Data from an additional four participants were excluded due to display change awareness (they reported seeing changes on > 5% of trials).

#### Stimuli and design

To achieve matching spatial width for both valid and invalid previews, we selected 60 verb forms from Boudelaa et al. (2019), each inflected for person, number, and gender. Invalid previews were created by transposing two internal letters, which preserved the overall spatial width of the word in Arial font. Specifically, the transposition involved the first two consonants of the root, while the remaining letters were unchanged. Each target word was used in two different sentences, presented in separate blocks, along with 20 filler sentences. Each participant encountered a total of six practice sentences, 120 experimental sentences, and 20 filler sentences.

The pre-target words consisted of 4 to 10 letters (*M* = 6.0), while all target words were 5 to 8 letters long (*M* = 6.2). Their frequency varied from 0.03 to 2.03 occurrences per million (*M* = 0.4, *SD* = 0.5) according to the Aralex database (Boudelaa & Marslen-Wilson, 2010). The target words were embedded within sentences that ranged from 8 to 12 words in length (*M* = 9.6), with the target word consistently appearing near the middle of the sentence.

To assess predictability, a cloze task was conducted involving 24 participants who did not take part in the eye-movement study. The results showed that the target words were not predictable from the sentence context (*M* = 0.4% correct completions, *SD* = 1.9%). To assess plausibility, another norming task was conducted with 24 participants. This task confirmed that the target words were highly plausible within the sentence context (*M* = 4.0, *SD* = 0.4, on a 5-point Likert scale, where 1 indicated highly implausible and 5 indicated highly plausible).

#### Apparatus

Eye movements were recorded using a tower-mounted EyeLink 1000 Plus (SR Research Ltd.) eye-tracker. Right eye gaze location was recorded each millisecond during binocular viewing. As in Experiment 1, sentences were displayed from a distance of 80 cm on a high-definition 24-inch BenQ monitor, with a 1,920 × 1,080-pixel resolution and a refresh rate of 144 Hz, using Arial and Kawkab-Mono fonts in the same font sizes used in Experiment 1.

#### Procedure

The experimental procedure was the same as in Experiment 1.

#### Analyses

Following the data-cleaning procedures described in Experiment 1, we first removed fixations shorter than 80 ms or longer than 1,200 ms (4.1% of fixations). We also cleaned the data to remove instances where the boundary change did not trigger as expected. We excluded trials in which a saccade triggered a display change but terminated to the left of the target word, or a display change was triggered by fixations before the boundary (i.e., a “j-hook”, accounting for 6.2% of trials). We also excluded cases where the display change completed more than 10 ms after the onset of the subsequent fixation (accounting for 2.2% of trials). Finally, we removed observations ≥ 2.5 SD from the mean of each participant in each condition, accounting for < 2.4% of observations for each measure). In total, a maximum of 10.8% of trials were removed during data cleaning. Eye-movement measures and model-fitting procedures were the same as in Experiment 1. For all analyses, participants and stimuli were treated as crossed random effects, and font type was treated as a fixed effect. Sentence-level analyses were restricted to the valid preview condition. For target-word analysis, preview validity plus all interactions between font type and preview validity were additionally included as fixed effects. Final converging model structures for target-word measures are listed in full below the corresponding statistical output tables.

### Results

Means and standard errors for sentence-level measures are shown in Table 1, with statistical output in the text. Means and standard errors for target-word measures are given in Table 4, and statistical output in Table 5.

**Table 4:** Means and standard errors (in parentheses) for target-word measures in Experiment 2

	Arial		Kawkab-Mono	
	Valid preview	Invalid preview	Valid preview	Invalid preview
Word skipping	.05 (.01)	.04 (.01)	.04 (.01)	.03 (.01)
Single-fixation duration	337 (17)	365 (17)	329 (15)	370 (20)
First-fixation duration	298 (13)	316 (11)	285 (11)	305 (11)
Gaze duration	399 (19)	428 (22)	392 (20)	431 (23)

**Table 5:** Target-word analyses for Experiment 2, showing fixed effect estimates (*b*), standard errors (*SE*), and *t*/*z*-values for preview validity (valid, invalid) and font (Arial, Kawkab-Mono) on word skipping, single-fixation duration (SFD), first-fixation duration (FFD), and gaze duration (GD). For convenience, estimates with an absolute value of < 0.01 are reported as 0.00

Fixed effect	*b*	*SE*	*t*/*z*	*p* value
Word skipping				
Intercept	− 3.99	0.24	− 16.79	< .001
Preview validity	0.00	0.22	0.00	1.00
Font	− 0.47	0.25	− 1.86	.064
Preview validity × font	0.41	0.38	1.07	.283
SFD				
Intercept	353.04	16.01	22.05	< .001
Preview validity	28.72	6.69	4.29	< .001
Font	− 2.45	7.70	− 0.32	.753
Preview validity × font	10.13	9.98	1.02	.310
FFD				
Intercept	301.32	10.74	28.07	< .001
Preview validity	18.41	4.46	4.13	< .001
Font	− 11.32	5.07	− 2.23	.031
Preview validity × font	2.06	11.15	0.19	.855
GD				
Intercept	413.7	20.26	20.39	< .001
Preview validity	33.61	8.24	4.08	< .001
Font	− 2.43	7.01	− 0.35	.731
Preview validity × font	8.77	15.80	0.56	.582

The relevant maximal structure for these models is (depvar ~ preview*font + (1 + preview*font |pp) + (1 + preview*font|stim). Final converging model structures were as follows: Skip = glmer(depvar ~ preview*font + (1 |pp) + (1 + preview + font|stim), data = datafile, family = binomial, control = glmerControl (optimizer = "bobyqa", optCtrl = list(maxfun = 100,000))); SFD = lmer(depvar ~ preview*font + (1 + preview + font |pp) + ( 1 + font||stim), data = datafile_SFD, control = lmerControl(optimizer = "bobyqa", optCtrl = list(maxfun = 100,000))); FFD = lmer(depvar ~ preview*font + (1 + preview * font |pp) + ( 1 |stim), data = datafile_FFD, control = lmerControl(optimizer = "bobyqa", optCtrl = list(maxfun = 100,000))); GD = lmer(depvar ~ preview*font + (1 + preview * font |pp) + (1 |stim), data = datafile_GD, control = lmerControl(optimizer = "bobyqa", optCtrl = list(maxfun = 100,000)))

#### Sentence-level analyses

There was no significant effect of font on sentence reading times (*b* = 43.99, *SE* = 38.41, *t* = 1.20, *p* = .239). When reading a monospaced font, fixation durations were shorter (*b* =  − 16.10, *SE* = 1.84, *t* =  − 8.74, *p* < .001), but the number of fixations was greater (*b* = 0.87, *SE* = 0.14, *t* = 6.19, *p* < .001). There was no difference in the average length of progressive saccades in characters, or the rate of regressions across the two font types (*t*s < 0.70, *p*s > .50).

#### Target-word analysis

**Fig. 4:**
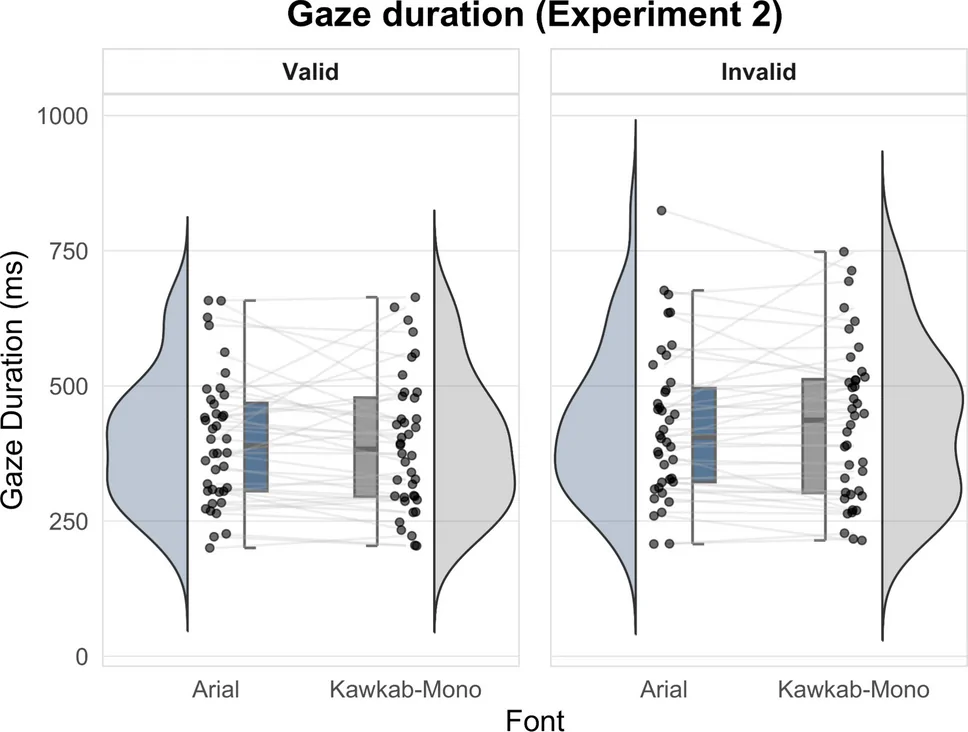
Raincloud plots of gaze duration (ms) in Experiment 2 as a function of font (Arial vs. Kawkab-Mono) and preview validity (valid vs. invalid). Each distribution shows the probability density (half-violin), median and interquartile range (boxplot), and individual participant means (dots). Gray lines connect the same participant across fonts within each preview condition

There was no significant effect of font type for SFD or GD, with similar fixation durations for both Arial and Kawkab-Mono. However, there was a significant effect of font in FFD, with shorter first fixations in the monospaced font than the proportional font. There were significant effects of preview validity across all reading time measures, with slower fixation times following invalid than valid previews (see Fig. 4 for GD). There were no significant effects of either font or preview validity on word skipping, with low skipping rates across all conditions (< 5%). Crucially, there was no evidence of an interactive effect between font and preview validity, indicating that participants obtained a similar level of preview benefit in the two font conditions.

### Discussion

In Experiment 2, sentence-level analyses replicated the pattern found in Experiment 1: overall reading times were equivalent for the two font types, but the monospaced font produced shorter fixation durations and more fixations than the proportional font. This again supports the notion of a trade-off between reduced crowding and increased width (Rayner et al., 2010). Experiment 2 also revealed that monospaced fonts yield shorter first-fixation durations than proportional fonts. One possibility is that this pattern relates to the greater length and morphological complexity of the Experiment 2 target words, although this cross-experiment comparison remains tentative (see Hautala et al., 2011, for similar effects of word length and complexity on eye movements).

Crucially, target-word measures showed a clear and consistent effect of preview validity for both monospaced and proportional fonts: Fixation durations were longer following an invalid preview, confirming reliable parafoveal processing. Importantly, there was no evidence of reduced parafoveal preview in a monospaced font, with preview benefits ranging from 18 to 29 ms in the proportional font and 20 to 41 ms in the monospaced font. This demonstrates that, despite the altered spatial relationships in monospaced fonts, readers are still able to effectively extract and use parafoveal information. Thus, monospaced fonts appear suitable for Arabic reading research, as they do not disrupt key aspects of normal reading.

## General discussion

We designed two experiments that examined the influence of font type (monospaced vs. proportional) on foveal (Experiment 1) and parafoveal (Experiment 2) processing during Arabic reading. These represent benchmark effects and so provide important test cases for evaluating the suitability of different font types for use in eye-movement research on reading. Taken together, these experiments produced a number of important findings that challenge assumptions about the unsuitability of monospaced fonts for research on Arabic reading, contributing to theoretical understanding of typographical influences on reading, and offering methodological advancement to support the design of Arabic reading experiments.

Despite concerns that monospaced fonts might disrupt the natural flow of the Arabic script, we found comparable reading performance across font types. However, monospaced fonts produced shorter fixations and a greater number of fixations, and in Experiment 1 (but not Experiment 2), progressive saccades traversed fewer letters. This pattern mirrors findings in Latinate languages for monospaced fonts (Rayner et al., 2010) and proportional fonts with increased inter-letter spacing (Perea et al., 2016a, 2016b). Therefore, the trade-off between letter clarity and spatial width observed in Latinate languages also extends to Arabic reading. Importantly, reading speeds were equivalent, indicating that these opposing effects cancel out. Minakata and Beier (2021) suggest that readers can flexibly adapt their reading style to the demands of different visual formats without impeding reading performance. Therefore, this pattern is consistent with an adjustment in oculomotor behaviour rather than a difference in overall processing efficiency, demonstrating that skilled Arabic readers adjust their eye-movement behaviour to accommodate typographic variation. These differences in eye-movement behaviour are consistent with those reported in Latinate scripts, offering no evidence that Arabic is uniquely disrupted by monospacing.

The overall pattern of lexical frequency effects was consistent across font types, supporting the methodological viability of using monospaced fonts in studies of word identification in reading. Both first-fixation and single-fixation durations showed robust effects of lexical frequency, indicating that readers were able to efficiently access frequency information in both fonts. Gaze duration showed a larger frequency effect in the monospaced condition, largely because high-frequency words were read numerically faster in monospaced font, whereas low-frequency words showed similar gaze durations across fonts. This pattern is relevant because it does not suggest disruption in the monospaced condition; rather, monospacing may provide a small processing advantage for familiar words. However, as this was limited to a single eye-movement measure, we prefer not to overinterpret it, and further work will be needed to establish the robustness of this pattern. Taken together, these findings provide little evidence that monospaced fonts materially impair lexical access; indeed, established lexical frequency effects remained similar in magnitude on monospaced and proportional fonts,

In addition, Experiment 1 also showed lower skipping rates on low-frequency than high-frequency words for both font types. Of note, Hermena et al. (2019) reported no frequency-related differences in skipping in Arabic, but their target words were long (8–11 letters) and morphologically complex, so were less likely to be skipped, while AlJassmi et al. (2021a) reported skipping effects for short, predictable morphologically simple words. The pattern observed here, with four-letter words, is broadly compatible with the notion that effects of word skipping may emerge under conditions that support parafoveal lexical processing. The role of lexical variables in Arabic word skipping remains an important question for future research.

The findings of Experiment 2 offer new insights into how font type influences parafoveal processing, an area that remains poorly studied even in Latinate scripts. In both monospaced and proportional fonts, valid previews led to shorter reading times than invalid previews across all fixation time measures, confirming robust parafoveal preview benefits. Despite the wider spatial width of words in the monospaced font causing letters to project further into parafoveal vision, readers were able to extract meaningful information prior to fixation, suggesting that the mechanisms supporting parafoveal processing operate effectively across font type, even when spatial layout differs. These results indicate that for Arabic, where visual and morphological demands are high, monospaced fonts do not disrupt the efficient extraction of upcoming word information. Methodologically, this supports the viability of using monospaced fonts in gaze-contingent paradigms, where the ability to match the spatial width of previews is essential.

Experiment 2 also found slightly shorter first-fixation durations for words in a monospaced font than in proportional text. This aligns with the general trade-off observed across both experiments, where monospaced fonts tend to produce shorter but more frequent fixations. As first-fixation duration reflects only the initial fixation on a word, it captures the effects of this trade-off on word recognition more directly. In Experiment 2, where words were longer and morphologically more complex, readers were more likely to make multiple fixations, allowing the shortening of the initial fixation to emerge more clearly. However, the increased likelihood of refixations offsets this benefit, resulting in equivalent gaze durations. Relatedly, in Experiment 1, where words were shorter and morphologically simpler, gaze durations were slightly shorter for high-frequency words in the monospaced font. Taken together, these patterns suggest that while the overall trade-off between spatial extent and letter clarity remains consistent, its relative balance may shift depending on word-level characteristics such as length, frequency, or complexity. Arabic, with its rich visual and morphological variability, offers a useful methodological opportunity for examining how font characteristics interact with linguistic properties to shape this trade-off. Importantly, while font type may modulate specific processing dynamics in both Arabic and Latinate languages, it supports broadly equivalent overall efficiency, reinforcing the methodological suitability of monospaced fonts for studying Arabic reading.

Returning to the claim that monospaced text in Arabic appears “extremely unnatural”, we may also consider reader perception of text, not just processing metrics. Hermena et al. (2017) did report that Courier New was dispreferred by readers. However, the most frequently “preferred” font in their study, Traditional Arabic, is a handwriting-style typeface that allows for vertical stacking of letters, a feature that is uncommon in digital media due to rendering incompatibility with Latin-script typesetting engines (W3C Internationalization Working Group, 2024). Operating systems such as Windows, Android, and iOS all use default fonts that do not allow for vertical overlap. This raises questions about what drives stated font preferences. Rather than reflecting ease of processing, such preferences may be shaped by social or cultural norms, or perceptions of formality and familiarity. Crucially, it remains unclear whether stated preferences correspond to differences in cognitive processing, and preference is rarely measured in studies using Latinate languages. Moreover, font exposure is highly variable across age groups, regions, and platforms. With increasing exposure to typographic diversity, particularly among digital-native readers, the salience of any one font format may be reduced (Minakata & Beier, 2021). Therefore, while it is possible that font preference influences reading, this remains an open question across all scripts, and there is no compelling reason to treat this as a unique barrier to using monospaced fonts in Arabic research when it has not been considered a barrier in Latinate-language research.

It is important to note that the monospaced font used in this study, Kawkab-Mono, was designed specifically for Arabic. It aims for an aesthetically pleasing appearance by combining elements of two traditional calligraphic styles: the smooth flow of Naskh and the angular geometry of Kufic (Arif, 2015). Compared with generic monospaced fonts commonly used in Latinate research, such as Courier New (the dispreferred font in Hermena et al., 2017) or Consolas, Kawkab-Mono may be perceived as more natural (Jordan et al., 2017). However, as discussed above, the relationship between font preference and reading behaviour remains poorly understood in both Arabic and Latinate languages. Thus, while Kawkab-Mono’s design characteristics may reduce its perceptual disruption, further research is needed to determine whether the present findings reflect general properties of monospaced fonts, or rather they are specific to this particular typeface. Addressing this question will be important for establishing the methodological generalizability of monospaced fonts in reading research.

The key issue for the present research, however, is whether monospacing affects core cognitive processes of reading. The present experiments suggest that it does not. In the two experiments, manipulating lexical frequency and preview validity, which represent benchmark effects in eye-movement research, we found consistent effects in fixation time measures, providing clear evidence that monospacing does not disrupt key cognitive processes underlying word recognition. This offers reassurance that monospaced fonts can effectively support investigations of word recognition in Arabic. The methodological foundation set out in the current paper may be expanded in important ways by future research. We focused on fixation durations, as these offer a direct index of lexical processing. We did not examine fine-grained saccade-targeting metrics such as launch site or landing position. Such measures are most informative in studies that manipulate word length or spacing, whereas our target words were length-matched and spatial layout was held constant across conditions. Nevertheless, previous findings in Arabic (Hermena et al., 2017) and English (McDonald, 2006) show that spatial extent affects saccade targeting. This highlights the importance of dissociating these dimensions. Fonts provide one way to do this. Because monospaced fonts offer a fixed letter-to-width ratio, they enable systematic manipulation of spatial extent and letter count. This creates opportunities to examine how visual layout influences saccade planning. Importantly, variation in saccade behaviour across font types need not imply different underlying mechanisms; rather, it may reflect the natural flexibility of the oculomotor system to respond to visual format (Minakata & Beier, 2021).

The present work also contributes to closing the cross-linguistic gap in eye-movement research on reading. Arabic is currently underrepresented in the eye-tracking literature, despite its unique visual and linguistic properties. New large-scale initiatives such as the Multilingual Eye-movement Corpus (MECO; Siegelman et al., 2022, 2025) are beginning to address this imbalance by extending comparable eye-movement datasets across a wider range of languages, including Arabic in the next iteration. These projects rely on matched linguistic materials across languages, meaning that any methodological artifact, such as font-type differences, could reduce comparability if not well understood. By demonstrating that monospaced fonts support robust lexical and parafoveal processing in Arabic, the current experiments provide a necessary methodological foundation to support the inclusion of Arabic in cross-linguistic reading research.

Overall, one thing is clear: we do not observe the level of disruption that would be anticipated if monospacing were “extremely unnatural”. Both monospaced and proportional fonts appear highly readable, and the observed differences largely replicate patterns reported in Latinate languages. This suggests that Arabic is not uniquely incompatible with monospaced fonts. Given the minimal disruption observed, the clear methodological advantages, and the potential to facilitate cross-language comparisons, it might be time to embrace monospaced fonts in Arabic reading research.

## Data Availability

The full dataset, experimental materials, and analysis scripts for both experiments are openly available on the Open Science Framework at https://osf.io/p5xhw/?view_only=af366c9353f1406184266ce51322f219.

## Footnotes

1 Removing random-effects terms because they could not be reliably estimated should not be taken to imply that their true variances are zero. If an omitted random slope has non-negligible variance, its removal can make tests of the corresponding fixed effect anti-conservative, thereby increasing the Type I error rate (Barr et al., 2013; Eager & Roy,. 2017). However, simulations reported by Matuschek et al. (2017) indicate that appreciable Type I error inflation is primarily a concern when random-slope variances are relatively large. Such variance components should generally be more strongly supported by the data and therefore less likely to be removed solely because the maximal model fails to converge. This risk is also less central to the present conclusions, which primarily concern the absence of clear font-related costs or interactions and are framed tentatively as a lack of evidence for these effects. Nevertheless, significant fixed effects from simplified models should be interpreted with the potential for anti-conservativeness in mind.
